# Functional and Morphological Improvement of Dystrophic Muscle by Interleukin 6 Receptor Blockade

**DOI:** 10.1016/j.ebiom.2015.02.014

**Published:** 2015-02-26

**Authors:** Laura Pelosi, Maria Grazia Berardinelli, Loredana De Pasquale, Carmine Nicoletti, Adele D'Amico, Francesco Carvello, Gian Marco Moneta, Angela Catizone, Enrico Bertini, Fabrizio De Benedetti, Antonio Musarò

**Affiliations:** a Institute Pasteur-Cenci Bolognetti, DAHFMO-Unit of Histology and Medical Embryology, IIM, Sapienza University of Rome, 00161, Italy; b Division of Rheumatology, Bambino Gesù Children's Hospital, Rome 00100, Italy; c Department of Neuroscience, Unit of Neuromuscular and Neurodegenerative Disease, Bambino Gesù Children's Hospital, Rome 00100, Italy; d Center for Life Nano Science@Sapienza, Istituto Italiano di Tecnologia, Rome 00161, Italy

**Keywords:** IL6, Muscular dystrophy, Inflammation, Necrosis, Therapy

## Abstract

The anti-inflammatory agents glucocorticoids (GC) are the only available treatment for Duchenne muscular dystrophy (DMD). However, long-term GC treatment causes muscle atrophy and wasting. Thus, targeting specific mediator of inflammatory response may be more specific, more efficacious, and with fewer side effects. The pro-inflammatory cytokine interleukin (IL) 6 is overproduced in patients with DMD and in the muscle of mdx, the animal model for human DMD. We tested the ability of inhibition of IL6 activity, using an interleukin-6 receptor (Il6r) neutralizing antibody, to ameliorate the dystrophic phenotype. Blockade of endogenous Il6r conferred on dystrophic muscle resistance to degeneration and alleviated both morphological and functional consequences of the primary genetic defect. Pharmacological inhibition of IL6 activity leaded to changes in the dystrophic muscle environment, favoring anti-inflammatory responses and improvement in muscle repair. This resulted in a functional homeostatic maintenance of dystrophic muscle.

These data provide an alternative pharmacological strategy for treatment of DMD and circumvent the major problems associated with conventional therapy.

## Highlights

• Inhibition of IL6 activity leads to changes in the dystrophic muscle environment.
• IL6R neutralizing antibody ameliorates the dystrophic phenotype.
• IL6 blockade counters muscle decline in mdx mice.

## Graphical abstract

**Figure:**
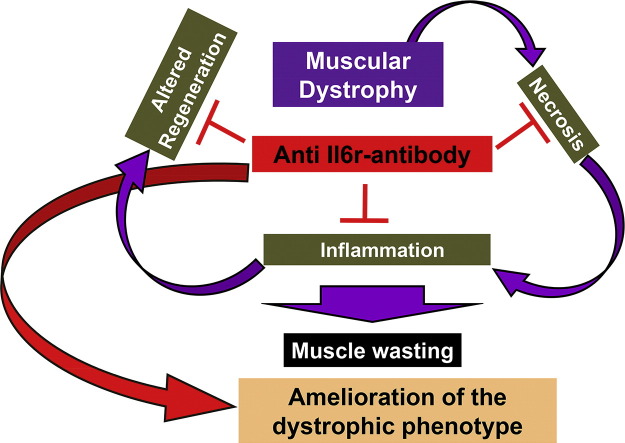

## 1 Introduction

Duchenne muscular dystrophy (DMD) is an X-linked genetic disease caused by mutations in the dystrophin gene that result in dysfunctional or absent dystrophin protein. The absence of dystrophin causes instability of the dystrophin–glycoprotein complex (DGC) with alterations in intracellular signaling leading to an imbalance between protein synthesis and protein degradation, with subsequent necrosis and fibrosis (Glass, 2007). The mdx mouse strain, with a point mutation within its *dystrophin* gene, has served as the animal model for human DMD (Carnwath and Shotton, 1987).

Although the mdx mouse presents some limitations compared to DMD patients, due to the fact that skeletal muscles of mdx mice undergo extensive necrosis only early in neonatal life, it remains an ideal model for preclinical tests and proof-of-concept studies (Bentzinger et al., 2014, Bogdanovich et al., 2002, Colussi et al., 2008, Consalvi et al., 2013, Denti et al., 2006, Grounds et al., 2008, Jiang et al., 2014, Kainulainen et al., 2015, Minetti et al., 2006, Price et al., 2014, Stupka et al., 2006, Tedesco et al., 2011, Vidal et al., 2012, Villalta et al., 2011, Villalta et al., 2014, von Maltzahn et al., 2012, Willmann et al., 2009). Different animal models, including the double mutant mice like mdx deficient for *MyoD1*, *utrophin*, *parvalbumin*, *alpha7 integrin*, or mTR (telomerase), have been generated and all of them share many phenotypical hallmarks with DMD (reviewed in Willmann et al., 2009). However, several aspects, including the genetic basis of the disease, must be considered when evaluating the animal models for use in the preclinical testing of potential new treatment options (Willmann et al., 2009). Thus, the mdx mouse, lacking a functional dystrophin gene, represents the most valid pre-clinical model, considering also that the double mutant mice do not resemble the genetic background of DMD patients and are therefore less appropriate to predict therapeutic effects (Willmann et al., 2009).

There is an acute onset of pathology (increased myofiber necrosis and elevated blood CK) around 3 weeks of age, in which mdx mice display muscle weakness similarly to DMD patients (Grounds et al., 2008) and the mdx muscles appear more susceptible to fatigue in vivo than control mice, similarly to other dystrophic models (Willmann et al., 2009).

Among factors involved in the pathogenesis of muscular dystrophy the extent of chronic inflammatory response has been suggested to be linked to the severity of dystropathology (Pescatori et al., 2007, Porter et al., 2002). Depletion of macrophages in the mdx mouse model at the early, acute peak of muscle pathology produced large reductions in lesions in the plasmalemma of muscle fibers (Wehling et al., 2001), showing that muscle macrophages that are present during the acute, degenerative stage of mdx dystrophy are highly cytolytic, and that they play a central role in the pathogenesis of muscular dystrophy.

Glucocorticoids, the most powerful anti-inflammatory and immunosuppressive agents available, are the only available treatment that allows to slow down disease progression in DMD patients (Horber and Haymond, 1990). However, it is well known from experience in chronic inflammatory diseases, not involving muscles, that long-term GC treatment causes muscle atrophy secondary to protein catabolism and muscle proteolysis (Horber and Haymond, 1990). Therefore, the efficacy of GC treatment in DMD patients is the net benefit of positive effects (suppression of inflammation) and negative effects (muscle catabolism). A better understanding of the inflammatory process in the dystrophic muscle and of the mediators involved might open alternative therapeutic perspectives.

In the present study we have focused our attention on the inflammatory cytokine interleukin 6 (IL6), based on the evidence that it is highly expressed in DMD patients and in mdx mouse model and it also plays a major role in inducing the transition from an acute neutrophilic infiltrate to a chronic type mononuclear cell infiltrate (Gabay, 2006).

IL6 is a pleiotropic cytokine that can contribute to the positive regulation of muscle homeostasis under physiological conditions and to the negative regulation of the muscle phenotype under some pathological circumstances (Fuster and Walsh, 2014, Munoz-Canoves et al., 2013). The complex actions of IL6 may be linked to the different manners by which this cytokine signals at the plasma membrane and by the different signaling pathways that can activate (Fuster and Walsh, 2014, Munoz-Canoves et al., 2013, Pedersen and Febbraio, 2008). Based on the activation of either classic or trans-signaling, IL6 can promote markedly different cellular responses. IL6 trans-signaling, which requires the soluble IL6R (sIL6R), is pro-inflammatory, whereas classic IL6 signaling, mediated by membrane-bound receptor, promotes regenerative or anti-inflammatory activities of the cytokine (Rose-John, 2012).

Circulating IL6 levels are normally very low or undetectable and are markedly increased in several diseases associated with inflammation, inducing the transition from an acute to a chronic inflammatory response (Gabay, 2006). IL6 is also locally and transiently produced in response to exercise and injury, and it plays an important role in satellite cell proliferation and muscle growth (Kurek et al., 1996, Serrano et al., 2008). In contrast, increased muscle proteolysis was found after administration of high doses or long-term exposure to IL6 in rodents (Goodman, 1994, Haddad et al., 2005, Tsujinaka et al., 1995). We recently demonstrated that treatment of C2C12 myogenic cells with recombinant IL6 resulted in a dramatic inhibition of myoblast differentiation (Pelosi et al., 2014). Interestingly, the inhibition of IL6 activity in an injured muscle, using an anti-interleukin-6 receptor antibody, promotes muscle regeneration via immune modulation (Fujita et al., 2014), whereas the inhibition of the IL6 intracellular mediator, namely the Jak/Stat pathway, stimulates muscle regeneration in both aged and dystrophic mice (Price et al., 2014, Tierney et al., 2014). The opposite effects exerted by IL6 can be justified considering the concept of hormesis, in which a low dose of a substance/molecule is stimulatory/homeostatic and a high dose is inhibitory/catabolic. Moreover, the positive effects of IL6 are normally associated with its transient production and short-term action. In contrast, persistent inflammatory conditions, including muscular dystrophy, are associated with long-lasting elevated systemic IL6 levels. Thus, muscle tissue might benefit from low and transient increases in IL6, whereas it is damaged by exposure to persistently high levels of this cytokine (Scott et al., 1996).

In the current study, we investigated whether specific blockade of IL6 activities may represent a potential therapeutic to treat human DMD. We revealed that blockade of IL6 activity in mdx mice, using a neutralizing antibody against the IL6 receptor (moAb-Il6r), confers robustness to dystrophic muscle, impedes the activation of a chronic inflammatory response, significantly reduces necrosis, activates the circuitry of muscle differentiation and maturation. This results in a functional homeostatic maintenance of dystrophic muscle.

## 2 Materials and Methods

### 2.1 Mice

Wild type and mdx mice were maintained according to the institutional guidelines of the animal facility of the unit of Histology and Medical Embryology. Mdx male mice (C57BL10) (purchased from Jackson Laboratories) were injected subcutaneously, starting at 15 days of age, with control IgG1 or with the neutralizing monoclonal antibody MR16-1 (kindly provided by Chugai Pharmaceutical Co., Ltd) (Okazaki et al., 2002) to the murine Il6 receptor at a dose of 100 μg/g of body weight and then twice a week with 20 μg/g (for a total of 5 doses) in PBS. Mice were sacrificed at day 30 of age. All animal experiments were approved by the ethics committee of Sapienza University of Rome-Unit of Histology and Medical Embryology and were performed in accordance with the current version of the Italian Law on the Protection of Animals.

### 2.2 Patients

All DMD patients had a molecular genetic detection of a mutation in the dystrophin gene predicting a marked dystrophin-deficiency. Sera from 29 non-treated DMD patients, 22 DMD patients treated with glucocorticoids and from 26 age-matched healthy controls were also collected. The human studies have been reviewed by the ethics committee of Bambino Gesù Children's Hospital and have therefore been performed in accordance with the ethical standards laid down in an appropriate version of the 1964 Declaration of Helsinki. All persons gave their informed consent prior to their inclusion in the study.

### 2.3 Treadmill Tests

The treadmill tests were performed using the LE8700 Treadmill Control (Panlab sl) (Cassano et al., 2008). Four week old mdx and moAb-Il6r-treated mice were acclimated to treadmill running (5 m/min for 5 min, after which the speed was increased 1 m/min every 2 min up to 9 m/min) before the test was performed. The mice (minimum 4 animals/strain) ran on the treadmill at an inclination of 0° at 5 m/min for 5 min, after which the speed was increased 0.6 m/min every 1 min. The test was stopped when the mouse remained on the shocker plate for more than 20 s without attempting to re-engage the treadmill, and the time to exhaustion was determined. The test was repeated four times (every two days).

### 2.4 Evans Blue Staining and Confocal Microscopy

Intraperitoneal injection of Evans blue dye (EBD) (100 μl of 1% EBD per 10 g of body mass) was performed on a minimum of 5 animals/strain (wild type, moAb-Il6r-treated mdx, and IgG1-treated control mdx mice). Fluorescent fibers were viewed under an inverted microscope (Axioskop 2 plus; Carl Zeiss Microimaging, Inc.), and images were processed using Axiovision 3.1 and analyzed using Scion Image 4.0.3.2. software. Confocal microscopy (Leica Laser Scanning TCS SP2) was used to analyze the total intensity of EBD fluorescence, which represents the full amount of fluorescence held within the entire z-axis of the series, and the mean amplitude of fluorescence in mdx and moAb-Il6r diaphragm muscles. Approximately 240 optical sections, from at least three separate experiments, were analyzed.

### 2.5 Immunofluorescence Analysis

Frozen human muscle biopsy sections were fixed in ice-cold acetone. Tissue sections were incubated 2 h at 37 °C with a mouse anti-human antibody against IL6 (R&D Systems, USA). The secondary antibody against mouse IgG, fluorescein conjugated (Chemicon, USA) was applied and incubated for 1 h at room temperature. Sections were also labeled with a polyclonal rabbit anti-human antibody against nidogen (Calbiochem) and then with the appropriate secondary antibody labeled with Alexa Fluor 555 (Invitrogen, Life Technologies, USA) 1 h at 37 °C.

### 2.6 Protein Extraction, Western Blot Analysis, ELISA, and Antibody Array

Diaphragm muscles from at least 5 animals/strain (wild type, mdx, and moAb-Il6r-treated and untreated mdx mice) were homogenized in modified lysis buffer (Tris–HCl, pH 7.5/20 mM, EDTA/2 mM, EGTA/2 mM, sucrose/250 mM, DTT/5 mM, Triton-X/0.1%, PMSF/1 mM, NaF/10 mM, SOV_4_/0.2 mM, cocktail protease inhibitors/1 × (Sigma) and processed for western blot analysis. Filters were blotted with antibodies against: pStat3 (Tyr705) (cat #9145, Cell Signaling), Stat3 (cat # 9132, Cell Signaling); NFkBp65 (ser536) (cat # 3033, Cell Signaling); and NFkB (cat #4764, Cell Signaling). Signals were captured by ChemiDoc-It® Imaging System (UVP, LLC) and densitometric analysis was performed with VisionWorks®LS Image Acquisition and Analysis Software (UVP, LLC). ELISA assay was performed using either a human (to detect IL6; cat # HS600B) or mouse (to detect Il6, cat # M6000B, and Tnfα, cat # MTA00B) Quantikine® Colorimetric Sandwich ELISAs (R&D Systems), according to manufacturer's protocol. Il2 expression was evaluated on the diaphragm of at least 3 animals/strain and with 2 biological replicates, using a RayBio® Mouse Antibody Array-G series (RayBiotech, Inc.), according to manufacturer's protocol. The intensities of signals were quantified with RayBio® Antibody Array Analysis Tool software.

### 2.7 RNA Extraction and Quantitative RT-PCR

Total RNA extraction was performed with tissue lyser (QIAGEN) in TriRiagentTM (Sigma). To synthesize single-stranded cDNA, 10 ng of total RNA was reverse transcribed using the TaqMan® MicroRNA Reverse Transcription Kit (Applied Biosystem), while double-stranded cDNA was synthesized with the QuantiTect Reverse Transcription kit (Qiagen). miRNA and mRNA analyses were performed on an ABI PRISM 7500 SDS (Applied Biosystems), using specific TaqMan assays (Applied Biosystems). Relative quantification was performed using endogenous controls U6 snRNA for miRNAs and Hprt1 for mRNAs (Applied Biosystems, USA). The relative expression was calculated using the 2 − DDCt method.

### 2.8 Statistical Analysis

Statistical analysis was performed with GraphPad Prism Software. All data, if not differently specified, were expressed as mean ± SEM. Difference among groups were assessed with one-way ANOVA with a Bonferroni post-test, or Dunn's post-test and between pairs with Mann–Whitney test or Student's t test assuming two-tailed distributions. Sample size was predetermined based on the variability observed in preliminary and similar experiments. All experiments requiring animal models were subjected to randomization based on litter. Investigators were not blinded to group allocation or outcome assessment. p < 0.05 is considered statistically significant.

#### 2.8.1 Role of Funding Source

The study was mainly supported by Telethon (GGP13013) and partly by AFM, PRIN, and ASI. The funders had no involvement in the design, collection, analysis, interpretation, writing or decision to submit the article.

## 3 Results

### 3.1 IL6 Levels are Significantly Increased in DMD Patients and mdx Mouse Model

**Fig. 1:**
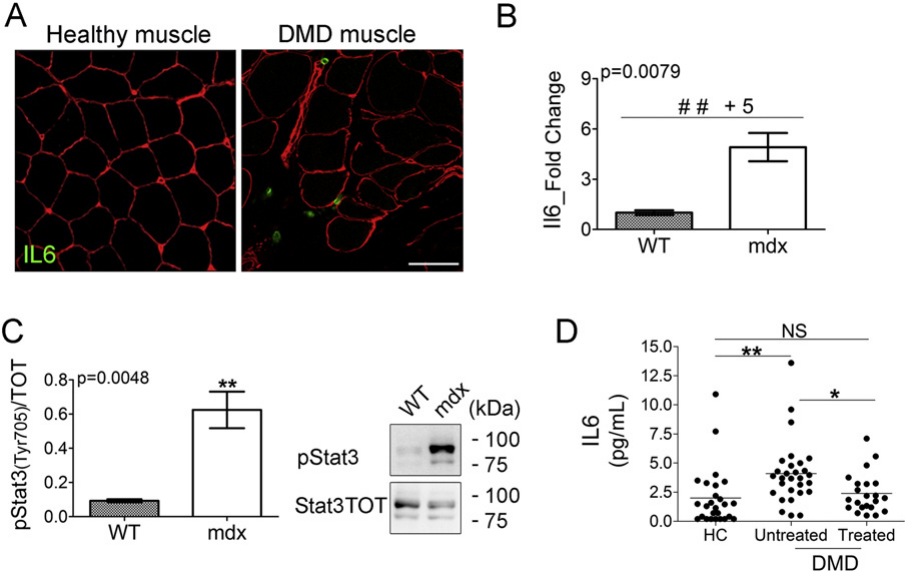
IL6 levels are accumulated in DMD patients and mdx mouse model. (A) Immunofluorescence staining of healthy human (left panel) and DMD (right panel) muscle biopsies immunolabeled with a rabbit anti-human nidogen (red) together with a mouse anti human IL6 (green). Images are representative of 29 experiments. Scale bar, 50 μm. (B) Expression of Il6 was evaluated by real time PCR in diaphragms of indicated genotypes at 4 weeks of age. Values represent mean ± SEM; n = 5. p value by Mann–Whitney test. (C) Western blot analysis (right panel shows a representative image) for active phospho-Stat3 (Tyr705) and total Stat3 protein performed on diaphragm muscles of wild type (WT) and mdx mice of 4 weeks of age. Values represent mean ± SEM; n = 6 to 9 per group. p value by Mann–Whitney test. (D) IL6 serum levels in healthy controls, untreated DMD patients and in DMD patients treated with corticosteroids. Values represent medians. n = 22 to 26 per group; NS = not significant. *p = 0.005; **p = 0.0001 by Mann–Whitney Rank Sum test.

In this study, we tested the effect of therapeutic inhibition of IL6 receptor (Il6r) to ameliorate the dystrophic phenotype in young (4 weeks of age) mdx mice, based on the evidence that IL6 protein expression was detected in infiltrating cells in the interstitial space among muscle fibers of DMD patients (Fig. 1A) and was significantly up-regulated in the diaphragm muscle of dystrophic mdx mice (Fig. 1B). The up-regulation of Il6 in the muscle of mdx mice was also associated with the increased expression of phosphorylated/active form of Stat3 (Fig. 1C), a molecular target of activated Il6 signaling (Heinrich et al., 1998). Moreover, circulating IL6 levels were significantly increased in DMD patients before initiation of treatment with glucocorticoids (GCs), compared to age matched controls and to DMD patients who were receiving GC treatment (Fig. 1D).

These data suggest that elevated levels of IL6 in DMD patients and in mdx dystrophic mouse model might contribute to exacerbate the dystrophic phenotype, sustaining an inflammatory response. Thus, it is conceivable to hypothesize that a blockade of IL6 signaling represents an approach for treatment of DMD. We therefore tested the hypothesis that the inhibition of Il6 effects in the mdx mice, using a neutralizing antibody against the Il6 receptor (moAb-Il6r) would lead to an amelioration of the muscle and functional phenotype.

### 3.2 Inhibition of Il6 Activity Counteracts Necrosis and Improves Functional Performance of Dystrophic Muscles

**Fig. 2:**
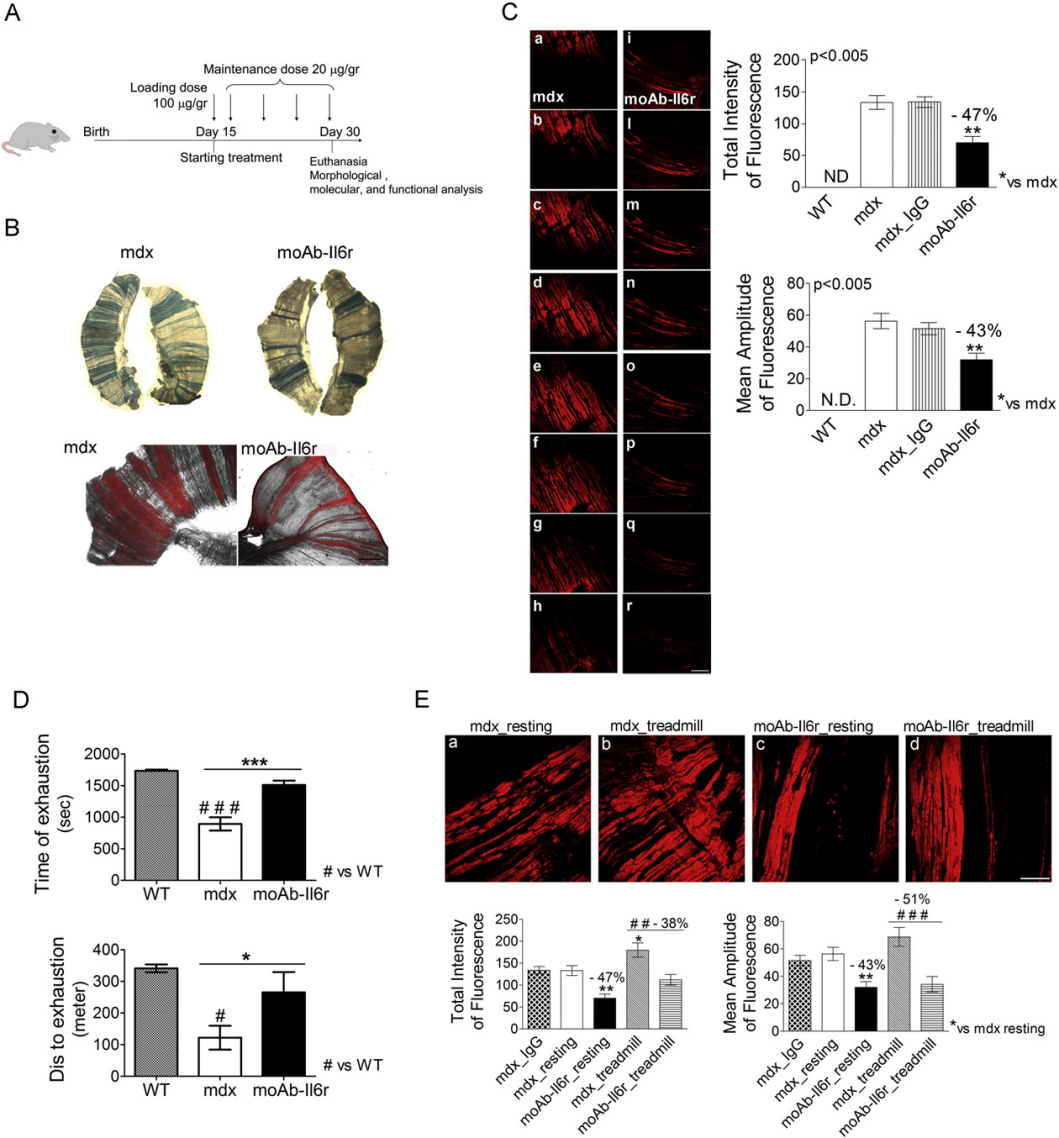
Il6 blockade confers on dystrophic mdx muscle resistance to degeneration and ameliorate dystrophic phenotype. (A) Schematic representation of moAb-Il6r treatment protocol in 15-week-old mdx mice. MoAb-Il6r was administered at an initial dose of 100 μg/g of body weight and then mdx mice were treated twice a week with 20 μg/g (for a total of 5 doses) in PBS. Mice were sacrificed at day 30 of age. (B) Representative images of whole diaphragm, stained with Evans blue dye (EBD), from moAb-Il6r treated and untreated mdx mice of 4 weeks of age. Necrotic region is blue in visible microscopy (top panels) and red in fluorescence microscopy (bottom panel). Scale bar, 500 μm. (C) Confocal microscopy: representative images of one spatial series (from 0 to 80 μm) composed of 8 optical sections with a step size of 10 μm, from whole diaphragms of 4-week-old mdx and moAb-Il6r-treated mdx mice. EBD is more diffuse in the diaphragm of mdx mice (a–h) than the diaphragm of moAb-Il6r-treated mdx mice (i–r). Scale bar, 150 μm. Graphs showing quantification of EBD uptake. Values represent mean ± SEM; n = 5 to 9 per group. p values, using one way ANOVA. *ND*, no detected. (D) Exhaustion treadmill tests carried on wild-type (WT), untreated, and moAb-Il6r-treated mdx mice. Note that moAb-Il6r-treated mdx showed increased running performance compared to untreated mdx mice. Muscle strength of treated and control animals was measured as time (top histogram) or distance to exhaustion (bottom histogram). Values represent mean ± SEM; n = 4 per group. *p < 0.05, ***p < 0.0005, ^#^p < 0.05, and ^###^p < 0.0005 using one way ANOVA. (E) Representative images of confocal analysis (top panels) and quantification of EBD uptake (bottom panels) performed on 240 optical sections from whole diaphragm muscles of indicated genotypes. Scale bar, 150 μm. Necrotic fibers evaluated by total intensity and mean amplitude of fluorescence (bottom) are significantly reduced in moAb-Il6r exercised mdx mice compared to mdx exercised mice. Values represent mean ± SEM; n = 5 to 9 per group. **p < 0.005, *p < 0.05, ^###^p < 0.0005, and ^##^p < 0.005 using one way ANOVA.

In order to investigate a possible therapeutic approach based on Il6 antagonism, we verified whether neutralization of Il6 activity ameliorated the dystrophic phenotype. Mdx mice were treated at 15 days of age with a neutralizing monoclonal antibody to the Il6 receptor (moAb-Il6r-treated), or IgG alone (control IgG-treated) (Fig. 2A). Mice were sacrificed at 4 weeks of age, at the stage when the acute manifestations of the disease in the mdx mouse model are clearly evident (Grounds et al., 2008).

We investigated the integrity of sarcolemma, measured by the percentage of necrosis, analyzing Evans blue dye (EBD) uptake in whole mount diaphragm muscles. We observed a marked reduction of EBD uptake in moAb-Il6r-treated mdx mice compared to untreated and control IgG-treated mdx mice (Fig. 2B and C).

It is known that dystrophic muscle is sensitive to mechanical stress, increasing myofiber necrosis and decreasing muscle strength (Brussee et al., 1997, De Luca et al., 2003, Okano et al., 2005). Treadmill running has been used to exacerbate the disease and to measure improvements of therapeutic approaches in mdx mice (Grounds et al., 2008). Although the treadmill test does not provide a direct evidence about muscle strength, it is a useful experimental tool to analyze the capability of dystrophic muscle to respond to mechanical damage induced by exercise (Burdi et al., 2006, Pierno et al., 2007). In fact, treadmill increases muscle necrosis in mdx mice, exacerbating the disease. Moreover, the in vivo weakness produced by such a protocol is observed exclusively in mdx mice with no similar effects in wild type mice; thus providing a reliable in vivo index with which to rapidly monitor potential drug efficacy (De Luca et al., 2002, De Luca et al., 2003, Granchelli et al., 2000). In fact, the ability of mdx mice to run on a treadmill (measured as distance run/day or week, or the time taken to cover a particular distance) is an indication of their general well-being and muscle function (Cassano et al., 2008, Dupont-Versteegden et al., 1994, Radley et al., 2008). Moreover, young mdx mice at the acute phase of dystropathology show muscle weakness and the mdx muscles appear more susceptible to fatigue in vivo than control mice (Grounds et al., 2008).

A treadmill test was performed in order to evaluate the effect of the inhibition of Il6 activities on muscle performance of dystrophic mice (Fig. 2D). This in vivo motility assay revealed that moAb-Il6r-treated mdx mice performed much better than untreated littermates, covering on average a longer distance in comparison to their untreated counterparts (Fig. 2D). Of note, the mdx untreated mice were exhausted after the trained session and remained prostrated in the cages for an extended period of time compared to moAb-Il6r-treated mdx littermates. Importantly, following treadmill exercise we observed significantly less muscle damage, evaluated by EBD uptake, in moAb-Il6r-treated mice in comparison to aged-matched untreated mdx mice (Fig. 2E). These data suggest that moAb-Il6r-treated mdx mice were more resistant to damage and displayed an increased muscular performance.

### 3.3 Inhibition of Il6 Effects Reduces the Inflammatory Response in Dystrophic Muscle

**Fig. 3:**
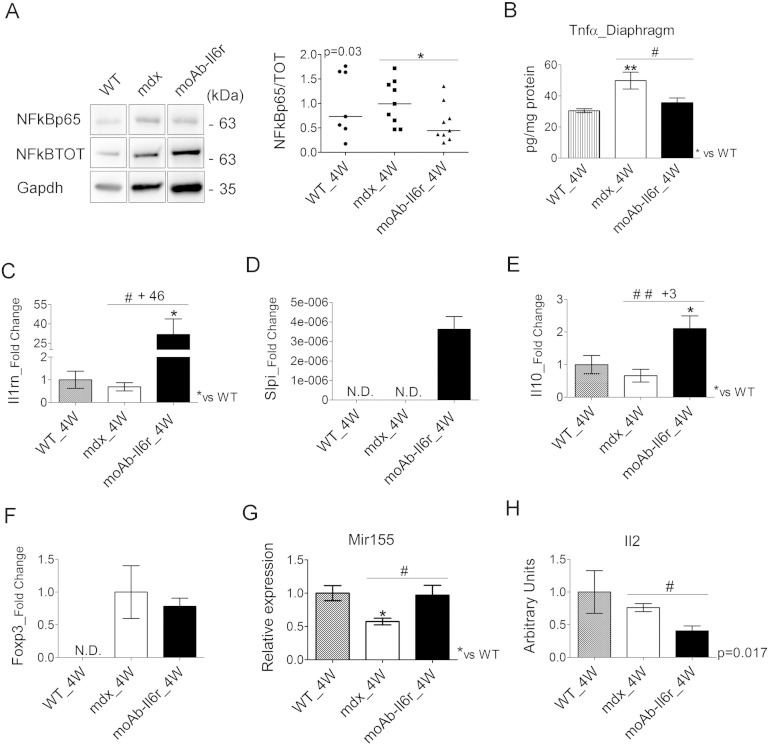
Il6 blockade modulates the inflammatory response. (A) Expression of NFkB (NFkB p65) active and total (NFkB TOT) proteins was evaluated by western immunoblotting (left panel shows a representative image) in the diaphragm of wild type (WT), moAb-Il6r-treated, and untreated mdx mice at 4 weeks (4 W) of age. Immunoblotting for Gapdh served as a control for protein loading. Graph shows quantification by densitometric analysis (right panel). Values represent medians. n = 7 to 9 per group. p value using Mann–Whitney Rank Sum test. In the left panel the lanes were run on the same gel but were non contiguous. (B) ELISA assay to evaluate the concentration (pg per mg of muscular protein) of Tnfα in the diaphragm of indicated genotypes at 4 weeks of age (4 W). Values represent mean ± SEM; n = 5 to 6 per group. **p < 0.005 and ^#^p < 0.05 using one way ANOVA. (C–G) Expression of Il1rn (C), Slpi (D), Il10 (E), Foxp3 (F), and Mir155 (G) was evaluated by real time PCR in diaphragms of indicated genotypes at 4 weeks (4 W) of age. Of note, Slpi expression was undetectable in the diaphragm of both 4-week-old WT and mdx mice, whereas Foxp3 expression was undetectable in the diaphragm of 4-week-old WT mice. Values represent mean ± SEM; n = 4 to 12 per group. *p < 0.05, ^#^p < 0.05, and ^##^p < 0.005 using one way ANOVA. ND, no detected. (H) Il2 expression, evaluated by RayBio® Mouse Antibody Array-G series (RayBiotech, Inc.), in diaphragms of wild type (WT), untreated mdx and moAb-Il6r-treated mdx mice at 4 weeks (4 W) of age. Values represent mean ± SEM; n = 3 to 4 per group. p value using t-test.

In order to evaluate whether reduced necrosis and exercise-induced damage in moAb-Il6r-treated mdx diaphragm were associated with changes in muscle inflammation, we analyzed the expression markers of the inflammatory response in moAb-Il6r-treated and untreated mdx mice. Interestingly, Il6 blockade resulted in a significant decrease in the synthesis and phosphorylation of NFkB protein (Fig. 3A) and in a marked down-modulation of Tnfα (Fig. 3B) expression. We also evaluated the expression of markers of the anti-inflammatory response (Tidball and Villalta, 2010, Villalta et al., 2011). We found an up-regulation of Il1rn (Fig. 3C), Slpi (Fig. 3D) and Il10 (Fig. 3E) in moAb-Il6r-treated mdx compared to untreated mdx mice.

One of the more interesting recent discoveries in DMD is that infiltrating regulatory T cells (Tregs) suppress muscle inflammation and muscle injury in this disease (Villalta et al., 2014). To evaluate the possibility that blocking Il6 alters T cell activity in dystrophic muscles, we analyzed relevant markers of Tregs, including Foxp3 expression (Fig. 3F). We did not observe any significant modulation in Foxp3 expression between moAb-Il6r-treated and untreated mdx mice (Fig. 3F). Nevertheless, we observed that Mir155, a positive regulator of Treg cell differentiation and homeostasis (Curtale et al., 2013, Kohlhaas et al., 2009, Lu et al., 2009), was strongly up regulated in moAb-Il6r-treated compared to untreated mdx mice (Fig. 3G). Moreover, we revealed a significant down regulation of the cytokine Il2, a strong modulator of Treg cells, in the diaphragm of moAb-Il6r-treated mdx mice compared to untreated mdx littermates (Fig. 3H), further supporting the evidence that blockade of Il6 interferes with the establishment of a chronic inflammatory response.

### 3.4 Il6 Blockade Improves the Homeostatic Maintenance of Dystrophic Muscle

**Fig. 4:**
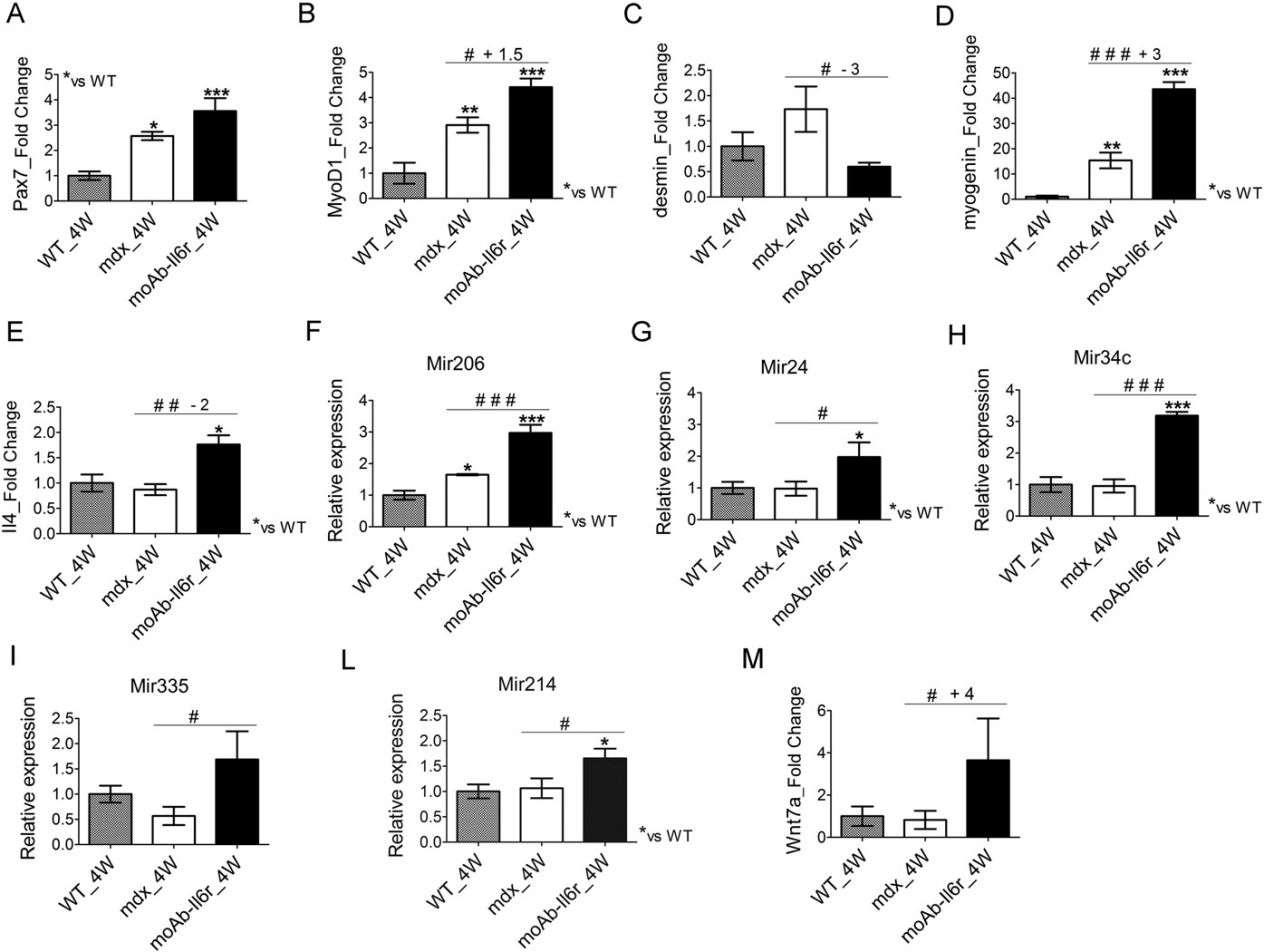
Il6 blockade improves the homeostatic maintenance of dystrophic muscle. (A–M) Expression of Pax7 (A), MyoD1 (B), desmin (C), myogenin (D), Il4 (E), Mir206 (F), Mir24 (G), Mir34c (H), Mir335 (I), Mir214 (L), and Wnt7a (M) was evaluated by real time PCR in diaphragms of wild type (WT), untreated mdx and moAb-Il6r-treated mdx mice at 4 weeks (4 W) of age. Values represent mean ± SEM; n = 4 to 6 per group. *p < 0.05, **p < 0.005, ***p < 0.0005, ^#^p < 0.05, ^##^p < 0.005, and ^###^p < 0.0005 using one way ANOVA.

The reduced necrosis and inflammation suggest a stabilization of dystrophic muscle phenotype with a reduced cyclic progression of necrosis and regeneration. These evidences were further supported by the analysis of relevant markers of activated, proliferating and differentiating satellite cells (Musaro, 2014, Rantanen et al., 1995). We found that Pax7 expression did not significantly change between untreated and moAb-Il6r-treated mdx mice (Fig. 4A), whereas MyoD1 expression was significantly up-regulated in moAb-Il6r-treated mdx compared to untreated mdx mice (Fig. 4B), suggesting that satellite cells are induced to differentiate. In fact, we observed a significant down regulation of desmin (Fig. 4C), a marker of proliferating satellite cells (Musaro, 2014, Rantanen et al., 1995), and a significant up-regulation of myogenin (Fig. 4D), a critical player for the terminal differentiation of myoblasts (Hasty et al., 1993, Musaro, 2014, Nabeshima et al., 1993). It has been demonstrated that Il4 acts as a myoblast recruitment factor during mammalian muscle differentiation and growth (Horsley et al., 2003). Real time PCR analysis revealed a significant up-regulation of Il4 expression in the muscle of moAb-Il6r-treated mdx compared to untreated mdx mice (Fig. 4E), suggesting that Il6r neutralization leads to changes in the dystrophic muscle environment, favoring anti-inflammatory responses and improvement in muscle repair.

To better define the improvement in the stabilization of muscle phenotype and muscle growth in anti-Il6r treated mice, we analyzed other relevant markers of muscle differentiation.

Interestingly, we observed an increase in Mir206 expression (Fig. 4F), a muscle specific miRNA activated by MyoD1, in moAb-Il6r-treated mdx compared to untreated mdx mice. Through Mir206 activation, MyoD1 might facilitate progression toward terminal differentiation (Chen et al., 2010, Hirai et al., 2010). Other key players that function during muscle regeneration, differentiation and homeostatic maintenance of skeletal muscle tissues are the non-muscle-specific Mir24, Mir34c, Mir335, and Mir214 (Eisenberg et al., 2009, Erriquez et al., 2013, Greco et al., 2009, Sun et al., 2008). We observed that Mir24, Mir34c, Mir335, and Mir214 were significantly up-regulated in the diaphragm of moAb-Il6r-treated mdx mice compared to untreated mdx littermates (Fig. 4G–M).

It has been reported that Wnt signaling promotes symmetric cell divisions by acting through the planar cell polarity pathway (Le et al., 2009). Specifically, Wnt7a, which is upregulated late in regeneration, promotes the symmetric expansion of satellite stem cells to replenish the stem cell pool and maintain a homeostatic level of stem cells through rounds of regeneration (Le et al., 2009). It has been also reported that Wnt7a treatment ameliorates muscular dystrophy, reducing the level of contractile damage and significantly increasing muscle strength of mdx mice (von Maltzahn et al., 2012).

Interestingly, we found an up-regulation of Wnt7a in the diaphragm of moAb-Il6r-treated mdx mice, compared with untreated mdx littermates (Fig. 4M).

These data suggest that the modulation of Il6 activities ameliorates muscle diseases in a dystrophic mouse model, without altering the induction of factors associated with muscle differentiation and growth.

To further reveal whether blockade of Il6 activity interferes with the myogenic program we treated C2C12, a muscle cell line derived from adult mouse satellite cells (Blau et al., 1985, Yaffe and Saxel, 1977), with Il6r-neutralizing antibody and we did not observe any significant alteration in the myogenic properties, including muscle proliferation and differentiation (data not shown).

## 4 Discussion

The major findings of this study indicate that inhibition of Il6 activity is a possible therapeutic strategy to counteract necrosis and the consequences of chronic inflammation in muscular dystrophy.

We found that Il6 is highly expressed in the plasma and muscle from patients with DMD and from young mdx mice. Thus, it is conceivable to hypothesize that a blockade of Il6 signaling represents an approach for treatment of DMD. We therefore tested the hypothesis that the inhibition of Il6 effects in the mdx mice, using a neutralizing antibody against the Il6 receptor (moAb-Il6r) would lead to an amelioration of the muscle and functional phenotype. Mdx mice at the pre-necrotic stage of the disease (i.e. 2 weeks of age) were treated and sacrificed at 4 weeks of age, which represents the stage when marked degeneration and regeneration of muscle fibers are observed. The choice to interfere with Il6 activity at the pre-necrotic stage of the disease in mdx mice was based on this rationale: a) treatment starts before the major initial bout of necrotic damage to mdx muscles occurs, b) analysis and interpretation of data are simplified due to the absence of pre-existing background tissue damage, and c) this young age relates to childhood events in human DMD, therefore making this approach more easily translatable to patients (Grounds et al., 2008).

Interestingly, the acute onset of myofiber necrosis provides a good model to study therapeutic interventions designed to prevent or reduce necrosis, since a reduction in dystropathology is easily identified (Grounds and Torrisi, 2004, Radley and Grounds, 2006, Shavlakadze et al., 2004, Stupka et al., 2001).

Our results demonstrate that inhibition of Il6 activities is beneficial to dystrophic muscle decreasing inflammation-induced myonecrosis, favoring the maintenance of muscle function, and preventing exercise-induced fiber damage and loss of muscle strength. These findings were paralleled by inhibition of NFkB expression and with modulation of the inflammatory response, including down-regulation of Tnfα and Il2 and upregulation of the anti-inflammatory mediators Il10 and Slpi.

It has been demonstrated that treated mdx mice with Il2/anti-Il2 complexes showed an increase in Il10 concentrations and suppression of type 1 inflammation (Villalta et al., 2014). Thus, our data demonstrated that blockade Il6 resulted in a significant decrease in the expression and activity of important mediators of the type 1 inflammatory response, associated with up-regulation of relevant markers of the anti-inflammatory response. This results in the establishment of a qualitative environment that might guarantee the homeostatic maintenance of muscle phenotype and therefore the delay in the progression of muscular dystrophy.

The improvement in the stabilization of muscle phenotype in anti-Il6r treated mdx mice was monitored analyzing relevant markers of muscle differentiation and maturation. Interestingly, we observed an up-regulation of factors that favor muscle differentiation and maturation, including myoD1, myogenin, Il4, Mir24, Mir206, Mir34c, Mir335, and Mir214. Interestingly, in addition to being strongly induced during myogenesis, Mir24 expression is maintained at high levels in terminally differentiated muscle tissues (Sun et al., 2008). Moreover, Mir34c and Mir335, along with Mir206, are up-regulated following myoblast differentiation in vitro (Greco et al., 2009), whereas Mir214 promotes cell cycle exit and skeletal muscle differentiation (Juan et al., 2009, Liu et al., 2010).

All of these data suggest that the maturation of the myogenic program and the homeostatic maintenance of dystrophic muscle tissues, which are severely affected by the absence of dystrophin expression, are facilitated by interfering with the pathologic activity of Il6.

Our results, conversely to those recently reported (Kostek et al., 2012), demonstrated that the methodologies employed, and the dose regimen of Il6r neutralizing antibody that has been used provide the rational for therapeutic approaches in human DMD based on IL6 inhibitors. Basically, several differences in the methodologies employed, including dosing regimen, and in the analysis and outcomes examined are present between the two studies. Although improvements were observed in the Kostek et al. study (Grounds and Torrisi, 2004), most of them did not reach statistical significance. Despite this, interestingly, and in agreement with molecular results of our study they found increased levels of Il10 in anti-Il6r treated mdx mice. This piece of data is in evident contradiction with their conclusion that inflammation is increased. It is reasonable to speculate that by inhibiting Il6, we reduced the inflammatory response in dystrophic mice and created a qualitative environment that makes the dystrophic muscle more resistant to the damage exerted by mechanical contraction.

Moreover, the recent reports indicating that inhibition of Stat3 activity, a downstream effector of Il6, enhances muscle repair and functional performance (Price et al., 2014, Tierney et al., 2014) strengthens our data and conclusion, considering that approaches based on the specific targeting of one inflammatory mediator may be more specific, more efficacious, and with fewer side effects, compared to glucocorticoids, as it is the case in chronic inflammatory diseases.

In conclusion, our work is consistent with a model in which moAb-Il6r-treatment confers robustness to dystrophic muscle, significantly reduces necrosis, impedes the activation of a chronic inflammatory response, activates the circuitry of muscle differentiation and maturation, guaranteeing a functional homeostatic maintenance of dystrophic muscle.

This might result in reduction in the cycle of regeneration and degeneration, which characterize the dystrophic untreated muscle, and therefore the need to continuously activate satellite cells. Duchenne muscular dystrophy is a disease of accelerated damage to muscle that causes the muscle stem cells (satellite cells) to eventually be used up. Thus, only treatments that stabilize the muscle so that continuous satellite cell repair is not needed will delay the progression of disease. Much of the damage comes from inflammatory response. Indeed, in the face of continuous inflammation, the satellite cells cannot properly repair the muscle and may even contribute to worsening of the disease.

The information derived by this study will have an impact on the immediate translation of IL6 blockade in the pharmacological treatment of DMD patients, since IL6 inhibitors are already available and their use have been recently approved in children with systemic juvenile idiopathic arthritis based on a favorable benefit/risk ratio (De Benedetti et al., 2012).
